# The impact of the COVID-19 pandemic on processes, resource use and cost in palliative care

**DOI:** 10.1186/s12904-023-01151-2

**Published:** 2023-04-06

**Authors:** Farina Hodiamont, Caroline Schatz, Eva Schildmann, Zulfiya Syunyaeva, Katerina Hriskova, Constanze Rémi, Reiner Leidl, Susanne Tänzler, Claudia Bausewein

**Affiliations:** 1 Department of Palliative Medicine, University Hospital, LMU Munich, Munich, Germany; 2 Helmholtz Zentrum München, Institute of Health Economics and Health Care Management, Munich, Germany; 3 Ludwig-Maximilians-Universität München, LMU Munich School of Management, Institute of Health Economics and Health Care Management, Munich, Germany; 4 Charité – Universitätsmedizin Berlin, corporate member of Freie Universität Berlin and Humboldt-Universität Zu Berlin, Department of Hematology, Oncology and Cancer Immunology, Oncological Palliative Care & Charité Comprehensive Cancer Center, Berlin, Germany; 5 Charité Universitätsmedizin Berlin, Department of Pediatric Respiratory Medicine, Immunology and Critical Care Medicine and Cystic Fibrosis Center, Berlin, Germany

**Keywords:** Costs, Resources, Covid-19, Quality of care, Palliative care, Pandemic, Cost comparison, Resource comparison

## Abstract

**Background:**

The COVID-19 pandemic impacts on working routines and workload of palliative care (PC) teams but information is lacking how resource use and associated hospital costs for PC changed at patient-level during the pandemic. We aim to describe differences in patient characteristics, care processes and resource use in specialist PC (PC unit and PC advisory team) in a university hospital before and during the first pandemic year.

**Methods:**

Retrospective, cross-sectional study using routine data of all patients cared for in a PC unit and a PC advisory team during 10–12/2019 and 10–12/2020. Data included patient characteristics (age, sex, cancer/non-cancer, symptom/problem burden using Integrated Palliative Care Outcome Scale (IPOS)), information on care episode, and labour time calculated in care minutes. Cost calculation with combined top-down bottom-up approach with hospital’s cost data from 2019. Descriptive statistics and comparisons between groups using parametric and non-parametric tests.

**Results:**

Inclusion of 55/76 patient episodes in 2019/2020 from the PC unit and 135/120 episodes from the PC advisory team, respectively.

IPOS scores were lower in 2020 (PCU: 2.0 points; PC advisory team: 3.0 points). The number of completed assessments differed considerably between years (PCU: episode beginning 30.9%/54.0% in 2019/2020; PC advisory team: 47.4%/40.0%). Care episodes were by one day shorter in 2020 in the PC advisory team. Only slight non-significant differences were observed regarding total minutes/day and patient (PCU: 150.0/141.1 min., PC advisory team: 54.2/66.9 min.). Staff minutes showed a significant decrease in minutes spent in direct contact with relatives (PCU: 13.9/7.3 min/day in 2019/2020, PC advisory team: 5.0/3.5 min/day).

Costs per patient/day decreased significantly in 2020 compared to 2019 on the PCU (1075 Euro/944 Euro for 2019/2020) and increased significantly for the PC advisory team (161 Euro/200 Euro for 2019/2020). Overhead costs accounted for more than two thirds of total costs. Direct patient cost differed only slightly (PCU: 134.7 Euro/131.1 Euro in 2019/2020, PC advisory team: 54.4 Euro/57.3 Euro).

**Conclusions:**

The pandemic partially impacted on daily work routines, especially on time spent with relatives and palliative care problem assessments. Care processes and quality of care might vary and have different outcomes during a crisis such as the COVID-19 pandemic. Direct costs per patient/day were comparable, regardless of the pandemic.

**Supplementary Information:**

The online version contains supplementary material available at 10.1186/s12904-023-01151-2.

## Background

Since January 2020, the COVID-19 pandemic impacts on all areas of society around the world, specifically with restrictions on social interactions. Health care systems are affected in manifold ways because of the challenging care of COVID-19 patients and the need to continue health care provision of other non-infected patients. Palliative care plays an important role during the pandemic in caring for patients infected with COVID-19 by supporting symptom management, decision making, including triage, and psychological support for patients and their families [1]. However, palliative care also needs to continue to support seriously ill and dying people without Covid-19 infection. Studies demonstrate the consequences of the pandemic on the care of seriously ill and dying people without Covid-19 infection. Mitchell et al. report the increased need for end-of-life care with higher numbers and more complex patients, especially for outpatients in the UK [2]. Because of infection prevention regulations, daily routines changed for health care workers and patients, with less face-to-face contacts and increasing demand for remote consultations [3–5]. Health care services (such as hospices and long-term-care facilities) in Germany did temporarily not accept any new patients, which might have contradicted continuity of care. The situation in specialized palliative care services, such as palliative care units, was also inconsistent, and ranged from the continuation of routine care under certain specifications to the closing of palliative care units or their rededication to Covid-19 units. The redistribution of palliative care professionals to Covid-19 units as well as measures to prevent infections and absence of personnel due to infections were reported to lead to staff shortages [5]. Assuming that the demand for palliative care did not decrease in the pandemic, the pandemics’ consequences might have a major impact on work routines and workload of palliative care teams. This assumption is supported by research showing that staff shortages increase staff’s perception of being more busy, compared to caring for patients before the pandemic [6] and that responsibilities and workload changed during the pandemic [4].

While research gives some insights in palliative care staff’s experiences and their perception of workload and work routine in palliative care during the pandemic, there is an evidence gap on how the pandemic affects resource use and associated hospital costs for palliative care at patient-level. However, this information is necessary to prepare for future care – in the acute COVID-19 pandemic situation as well as in other potential humanitarian crises in the future—both nationally and internationally.

Therefore, the aim of this study is to describe the differences in patient characteristics, care processes and resource use in specialist palliative care (palliative care unit and palliative care advisory team) in a university hospital before and during the first year of the COVID-19 pandemic.

## Methods

### Design

We conducted a retrospective, cross-sectional study using routine data of patients cared for in a palliative care unit and a palliative care advisory team as well as the cost data provided by the hospital’s accounting and personnel department. The “Strengthening the reporting of observational studies in epidemiology “ (STROBE) checklist was used to guide the reporting of this study [7].

#### Setting of the study

The study was conducted in the Department of Palliative Medicine at Munich University Hospital providing a ten bedded palliative care unit and a palliative care advisory team for the same hospital. In the palliative care unit, a multi-professional team consisting of a consultant, two registrars, 14 nurses, and a part-time social worker, physiotherapist, breathing therapist, psychologist and chaplain, respectively, cares annually for about 300 patients with advanced disease and at the end of their life. The department’s palliative care advisory team (one consultant, two registrars, two nurses with 1.5 posts full-time equivalent), part-time social worker and part-time psychologist) provides care for about 800 patients annually, who are treated across all departments of the University Hospital. The advisory team aims to advise primary carers in symptom management and goals of care discussions and support patients and families during the time of advanced disease focussing on improving their quality of life.

#### Study population

We included all patients who had an episode of care in one of the two specialist palliative care settings within the observation period. An ‘episode’ of care was defined as ‘a period of contact between a patient and a provider or team of providers that occurs in one setting’ [8]. Only completed episodes were considered for analyses – meaning that the episode was started and completed within the defined time period. Due to the course of the pandemic, the last quarter of the year 2020 (October – December) was chosen as observation period. This period was characterized by high infection and incidence rates all over Germany resulting in the respective safety measures in inpatient care including strict hygiene measures such as personal protective equipment but also restrictions and bans for visitors. On the normal hospital wards, clinically stable patients were not allowed any visitors. Patients at the end of life were granted one visitor per day for one hour. After the death of a patient due to COVID-19, the family members were not allowed to see the deceased due to infection control. On the palliative care unit, one visitor was allowed per patient for one hour per day throughout the first year of the pandemic. A negative test result for Sars-CoV-2 (PCR or antigen test) was required. At that time, no patients with COVID-19 were treated on the palliative care unit. If a patient was tested positive, the patient was transferred to a dedicated COVID-ward. Advice from the palliative care team for COVID-19 patients was only provided via telephone to reduce contacts and possible infection of health care workers.

For the reference year (2019), the same months (last quarter of the year) were chosen to exclude potential seasonal effects.

#### Data collection

Study data was extracted from the routinely collected data in the electronic patient records system used by the Department of Palliative Medicine. The data includes patient characteristics, information on care processes and labour time. All data were completely anonymised.

##### Patient characteristics

Age, sex, cancer or non-cancer diagnosis, professional assessment (either physician or nurse) of symptom burden at the beginning and end of the episode using the professional version of the Integrated Palliative care Outcome Scale (IPOS) which is a proxy version [9]. IPOS was reported by a) the total IPOS score (sum of all 17 item scores) with a value between 0 (not burdened at all) and 68 indicating an extremely high burden and palliative care needs, b) the physical symptom subscale score (sum of the 10 physical symptom items, possible score between 0–40), and c) the sum of the emotional symptom and communication/practical subscale score (sum of 7 items, score between 0–28) [9]. Only patient episodes with a complete IPOS assessment respectively a complete assessment of the IPOS subscale (symptom burden/psychosocial problems) were included in the analyses. Imputation methods were considered not to be applicable due to the uncertainty of reasons for missing assessments in possible dependencies on the pandemic situation.

##### Information on the episode of care

Length of care episode (date of admission to discharge/death), type of discharge (deceased/discharged to other care setting and information on respective care setting), number of professional groups involved in care (recorded via the professional ID in the documentation system, categorized by cases involving the minimum number or more professions as indicated by structural criteria for specialist palliative care in the German DRG-system in the respective setting, and cases involving less professions).

##### Information on labour time

Minutes of care spent on the overall care and proportionally on a) the level of individual occupational groups and b) in relation to care areas: patient (time spent in direct contact with the patient), relatives (time spent in direct contact with family members or friends), professional (time spent on patient-related contact and coordination with other team members/professionals), and systemic (time spent on activities regarding establishing or monitoring the treatment plan, e.g. documentation, reading information about the patient).

Labour time on direct patient care was documented differently in both settings. Each professional of the palliative care advisory team documented all their activities related to a patient in steps of 5 min in the electronic patient record. However, the documentation of time has its limitations on the palliative care unit. Nurses only documented palliative nursing specific activities in the departmental electronic patient record, e.g. specific wound care or conversations relevant to care planning. Time spent on general nursing activities, such as assisting with activities of daily living, e.g. body hygiene, food preparation, and general daily contacts with patients and their family, is not separately documented regarding the duration and are therefore excluded from this analysis.

##### Cost calculation

The cost calculation method was based on the framework of Mosoiu et al. and the main cost components identified by Gardiner et al. [10, 11]. Hospital unit-costs were calculated with top-down unit-cost rates for every patient and bottom-up collected resources, in minutes per day. Data from the accounting and personnel department, with prices from 2019, were applied for the top-down calculation. The unit-cost rates comprised all personnel costs from the palliative care unit and the advisory team, except research and teaching time of staff. These cost rates were multiplied with the bottom-up collected resources, in minutes, for each profession resulting in the direct cost components. Overhead personnel costs, as indirect costs, covered the remaining, not directly attributable minutes. Adapted from the framework of Mosoiu et al., length of stay at the respective setting was chosen as central allocation key for the overhead personnel costs [10]. Supplies and general hospital costs, and indirect costs, took into account all material costs from the palliative care team (e.g. medical consumables, drugs), and general costs (e.g. cleaning, meals). They were allocated with length of stay for the palliative care unit and per patient for the palliative advisory team. To account for the lack of documentation of nursing minutes spent on general nursing activities and their potential impact on the direct costs and the overhead costs, a sensitivity analysis, was conducted as a robustness check increasing the nursing minutes by one standard deviation.

### Statistical analysis

Descriptive statistics (frequency for categorical variables, median and interquartile range for continuous variables) were calculated for patient characteristics, care characteristics, and documented staff time. For categorical variables, Chi-Square test was used to detect significant differences between the two years (2019 and 2020). If expected cell frequencies were below 5, Fisher’s Exact test was performed. As most variables were not normally distributed, we used non-parametric (Mann–Whitney U-test) tests for continuous variables to calculate differences. A *p*-value < 0.05 was considered to be statistically significant. IBM SPSS Statistics 26 was used for analyses.

## Results

A total of 58 complete patient episodes were documented in the palliative care unit for the last quarter of 2019 and 78 episodes in 2020. Three and two outliers in terms of minutes spent on care were excluded in 2019 and in 2020, respectively (see boxplot in appendix 1), leaving 55 patient episodes for analysis in 2019 and 76 in 2020.

The palliative care advisory team documented 144 complete patient episodes during the study period in 2019 and 130 episodes in 2020. Data verification in the palliative care advisory team data set revealed some episodes with inconsistencies between the documented staff minutes (diverging documented minutes in categories of occupational groups and care areas, caused by a software problem in the saving process of the assessments). The respective episodes were removed from the data set (6 cases in 2019, 10 cases in 2020). Three more cases were removed from the 2019 sample as outliers in terms of minutes spent on care, leaving 135 episodes for analysis in 2019 and 120 in 2020.

In the palliative care unit, the median didn’t’ change during the pandemic compared to the year before. The mean length of care episodes was however 1.3 days shorter in 2020. Accordingly, more patients were cared for in the last quarter of 2020 (see Table 1). Similarly, the length of care episode of patients cared for by the palliative care advisory team was shorter by approximately one day in 2020 compared to 2019 (5.0 days in 2019 vs. 4.0 days in 2020, *p*-value = 0.422). In contrast to the palliative care unit, fewer patients were cared for by the palliative care advisory team in the last quarter of 2020 compared to the last quarter of 2019.

**Table 1:** Patient characteristics

	**Palliative Care Unit**							**Palliative Care advisory team**						
	**2019 (*N* = 55)**			**2020 (*N* = 76)**				**2019 (*N* = 135)**			**2020 (*N* = 120)**			
	**n**	**median/IQR**	**(%)**	**n**	**median/IQR**	**(%)/mean ∓ SD**	***p*-value**	**n**	**median/** **IQR**	**(%)/mean ∓ SD**	**n**	**median/IQR**	**(%)**	***p*-value**
Age	55	73.0 / 20.0		76	69.0 / 23.5		.492^b^	135	71.0 / 20.0		120	71.0 / 20.0		.797^b^
Sex							.673^c^							.279^c^
Male	32		58.2%	47		61.8%		80		59.3%	79		65.8%	
Female	23		41.8%	29		38.2%		55		40.7%	41		34.2%	
Main diagnosis cancer/non-cancer							.202^d^							.953^d^
Cancer	34		61.8%	55		72.4%		85		63.0%	75		62.5%	
Non-cancer	21		38.2%	21		27.6%		33		24.4%	31		62.5%	
No diagnosis documented	-		-	-		-		17		12.6%	14		62.5%	
Lenght of care episode (days)	55	8.0 / 7.0		76	8.0 / 9.0		.538^b^	135	5.0 / 5.0		120	4.0 / 4.0		.422^b^
IPOS at beginning of episode^a^	17	27.0 / 12	30.9%	41	25.0 / 10	54.0%	.166^b^	64	28.5 / 33.5	47.4%	48	25.5 / 11.5	40.0%	.059^b^
IPOS at end of episode	24	21.5 / 10.5	43.6%	37	20.0 / 13		.988^b^	58	28.0 / 34.0	43.0%	46	24.0 / 10.0	38.3%	**.049**^**b**^
IPOS symptom burden beginning	34	14.0 / 8	61.8%	57	10.0 / 8	75.0%	**.008**^**b**^	90	13.0 / 17.0	66.7%	76	12.0 / 9.0	63.3%	.167^b^
IPOS symptom burden end	41	12.0 / 7	(74.6%)	59	7.0 / 8	77.6%	**.014**^**b**^	84	13.0 / 18.0	62.2%	73	10.0 /8.0	60.8%	**.025**^**b**^
IPOS psychosocial problems beginning	22	13.5 / 5	40.0%	45	12.0 / 4	59.2%	.559^b^	71	14.0 / 16.0	52.6%	54	14.0 / 4	45.0%	.133^b^
IPOS psychosocial problems end	27	10.0 / 7	49.1%	44	11.5 / 5	57.9%	.440^b^	68	14.0 / 16.0	50.4%	53	13.0 / 3.0	44.2%	**.048**^**b**^
Number of SPC professions involved							.986^c^							.798^c^
1 -2 professions involved	-		-	-		-		82		60.7%	71		59.2%	
3–4 professions involved	-		-	-		-		53		39.3%	49		40.8%	
1–3 professions involved	16		29.1%	22		28.9%		-		-	-		-	
4–7 professions involved	39		70.9%	54		71.0%		-		-	-		-	
Type of discharge							.700^e^							.484^e^
Palliaitve Care Unit	-		-	-		-		35		25.9%	44		36.7%	
Home	6		10.9%	5		6.6%		38		28.1%	34		28.3%	
Hospital (other than PCU)	0		0%	2		1.5%		11		8.1%	8		6.7%	
Nursing home	3		5.5%	2		2.6%		2		1.5%	2		1.7%	
Hospice	4		7.3%	9		11.8%		-		-	-		-	
Deceased	40		72.7%	55		72.4%		34		25.2%	22		18.3%	
Other	2		3.6%	3		3.9%		15		11.1%	10		8.3%	

^a^Inclusion only of patient episodes with a complete IPOS assessment respectively a complete assessment of the IPOS subscale (symptom burden/psychosocial problems)

^b^*p*-values calculated with Mann–Whitney-U-test

^c^*p*-values calculated with Chi-squared test

^d^*p*-values calculated with Cramers-V

^e^*p*-values calculated with Fisher's Exact-test

Patient characteristics indicated slight differences comparing the two years (Table 1). Non-significant differences were detected comparing the two groups regarding age and sex (). The proportion of patients with non-cancer diagnoses decreased by 10.4% (38.2% in 2019 vs. 27.6% in 2020) on the palliative care unit, while no considerable difference was observed among the patients cared for by the palliative care advisory team. Differences were not significant (PCU: *p*-value = 0.202, PC advisory team: *p*-value 0 0.935).

On the palliative care unit, the median value of the IPOS total score on admission was 2 points lower in 2020 compared to 2019 (*p*-value = 0.166)). The median physical symptom scale score was significantly lower in 2020, both at the beginning (4.0 points difference, *p*-value = 0.008) and the end of the episode (5.0 points lower difference, *p*-value = 0.014). The psychosocial subscale showed no significant difference between the two years at both admission and the end of episode (1.5 points lower at admission and 1.5 higher at the end of episode;).

In patients cared for by the palliative care advisory team, the overall median IPOS score both, on admission and at the end of the care episode, was lower in 2020 compared to 2019: 3.0 points (*p*-value = 0.059) and 4.0 points (*p*-value = 0.049), respectively. Data for the latter was normally distributed. While the non-parametric test showed no significant difference for the IPOS-score at the beginning of the episode, the t-test was significant (*p*-value = 0.037). Looking at physical symptoms only, the symptom subscale score in 2020 was 1.0 points lower on admission compared to 2019 (not significant, *p*-value = 0.167) and 3.0 points lower at the end of the episode in 2020(*p*-value = 0.025). In 2020, the median psycho-social subscale score was the same at the beginning and 1.0 point lower at the end of the episode than the score in 2019 (*p*-value = 0.048).

Noteworthy in this context is the difference in completed IPOS assessments, respectively missings in the IPOS assessments, on overall and subscale level. On the palliative care unit, the number of completed assessments on overall and subscale level was considerably higher in 2020 than in 2019 while in the palliative care advisory team, it was the other way around. On admission to the palliative care unit, for example, only 30.9% (*n* = 17) of patients in 2019 had an overall IPOS assessment completed compared to 54% (*n* = 41) in 2020. In the palliative care advisory team, an overall IPOS assessment was completed for 47.4% (*n* = 64) at the beginning of an episode in 2019 and for 40.0% (*n* = 48) in 2020.

In total, more patients (*n* = 15) died on the palliative care unit during the pandemic observation period compared to the year before. The proportion of patients discharged to home, other hospitals, or nursing homes decreased while the proportion discharged to a hospice increased slightly (by *n* = 5, 4.5%). In patients cared for by the palliative care advisory team, 6.9% less died in 2020 compared to 2019, while the number of patients who were transferred to another hospital unit increased by 9.2%. Differences in both settings were not significant (PCU: *p*-value = 0.700, PC advisory team = 0.484).

In both settings, no differences regarding the categories of number of professions involved were observed (PCU: *p*-value = 0.986, PC advisory team: *p*-value = 0.484). The proportion of patient episodes in which less professions than the setting specific structural characteristic (below minimum number of involved professions/minimum or more professions involved) was almost identical in 2020 compared to the previous year.

No relevant difference was detected on the palliative care unit regarding the mean of total minutes per day and patient (see Table 2). The average minutes spent per day and patient for each professional group was only slightly different in most professional groups. The only statistically significant difference was seen for median staff minutes of physiotherapists who spent 3.8 min per day more with each patient in 2020 compared to 2019 (*p*-value = 0.006). Also, the number of minutes documented by the nursing staff decreased in 2020 from 95.0 min per day and patient to 92.0 min (*p*-value = 0.158). A more detailed breakdown of staff minutes by type of activity showed that the minutes spent per day on a particular activity area differed before and during the pandemic. The total number of minutes the team spent directly with the patient increased during the pandemic, while the number of minutes spent in direct contact with the family decreased significantly from 25.0 min in 2019 to 14.4 min per day in 2020 (*p*-value = 0.040). Looking only at documented minutes of nursing care, little difference was found in the daily time nurses spent with patients and on systemic activities. However, the time spent with patients’ families decreased significantly on average by 4.5% (6.6 min per patient and day, *p*-value = 0.007) during the pandemic and for professional contacts the time decreased by 1.7% (0.3 min per patient and day, *p*-value = 0.025).

**Table 2:** Documented staff minutes per patient and day palliative care unit

	**Palliaitve Care Unit**				
	**2019 (*N* = 55)**		**2020 (*N* = 76)**		
	**median / IQR**	**share in overall care (%)**	**median /IQR**	**share in overall care (%)**	***p*-value**
Minutes per patient and day	150.0 / 36.7	100%	141.1 / 50	100%	.539
Minutes per Patient and day by professional group					
Physician	28.8 / 17.4	22.5%	27.7 / 13.0	21.6%	.326
Nurse	95.0 / 34.0	66.5%	92.0 / 33.2	63.2%	.158
Social worker	1.8 / 4.5	2.4%	0.7 / 7.0	3.4%	.952
Respiratory Therapist	0.0 / 4.5	1.9%	1.0 / 7.5	2.4%	.150
Psychologist	0.7 / 5.0	2.0%	0.5 / 5.4	2.4%	.977
Physiotherapist	0.0 / 2.7	2.3%	3.8 / 9.7	3.7%	**.006**
Pastoral worker	0.7 / 5.2	2.4%	1.8 / 5.0	3.3%	.876
Minutes per Patient and day by field of activity (all professional groups)					
Patient	101.7 / 24.9	66.0%	103.3 / 34.1	70.8%	.446
Family	25.0 / 30.7	19.8%	14.4 / 24.6	15.9%	**.040**
Systemic	13.2 / 7.4	8.5%	11.8 / 5.8	9.0%	.853
Professional	6.7 / 7.1	5.8%	5.5 / 5.7	4.4%	.474
Nursing minutes per patient and day by field of activity					
Patient	77.9 / 21.0	78.9%	78.3 / 34.5	85.4%	.875
Family	13.9 / 16.1	17.6%	7.3 / 14.5	13.1%	**.007**
Systemic	1.0 / 2.0	1.3%	0.7 / 1.6	1.0%	.199
Professional	0.3 / 2.4	2.2%	0.0 / 0.6	0.5%	**.025**

*p*-values calculated with Mann–Whitney-U-test

Regarding the palliative care advisory team (Table 3), there were only slight non-significant differences in total minutes per patient per day between the years (*p*-value = 0.533). The situation was similar when minutes were broken down by professional group. However, similar to the data from the palliative care unit, there was a shift in staff minutes at activity level in the palliative care advisory team. The time spent in direct contact with patients’ family decreased significantly by 7.7% (by 1,5 min per day and patient, *p*-value = 0.019), while the time spent in communication with other professionals increased significantly by 7.7% (by 5.9 min per day and patient, *p*-value < 0.001).

**Table 3:** Documented staff minutes per patient and day palliative care advisory team

	**Palliative Care advisory team**				
	**2019 (*N* = 135)**		**2020(*N* = 120)**		
	**median / IQR**	**share in overall care (%)**	**median / IQR**	**share in overall care (%)**	**p-value**
Minutes per patient and day	54.2 / 60.1	100%	66.9 / 56.4	100%	.533
Minutes per Patient and day by professional group					
Physician	26.7 / 30.8	50.2%	29.4 / 33.3	53.7%	.634
Nurse	15.0 / 33.0	35.0%	15.0 / 29.9	33.1%	.968
Social worker	0.0 / 3.3	8.0%	0.0 / 10.4	9.1%	.441
Respiratory Therapist	0.0 / 0.0	0.2%			.0500
Psychologist	0.0 / 0.0	2.0%	0.0 / 0.0	0.9%	.200
Pastoral worker	0.0 / 3.3	4.7%	0.0 / 1.5	2.9%	1,000
Minutes per Patient and day by field of activity					
Patient	16.5 / 20.6	28.4%	17.2 / 19.2	28.6%	.680
Family	5.0 / 18.7	16.4%	3.5 / 9.7	9.7%	**.019**
Systemic	19.3 / 17.1	33.7%	19.2 / 17.7	32.7%	.846
Professional	10.8 / 10.3	21.4%	16.7 / 16.1	29.1%	**.000**

*p*-values calculated with Mann–Whitney-U-test

T-test sensitivity analyses for not equally distributed variables revealed no differences in significance compared with the non-parametric tests.

Total unit-costs per patient per day for the palliative care unit resulted in 1075 Euro for 2019 and 944 Euro for 2020 (see Table 4). Total unit-costs per patient per day were 161 Euro for 2019 and 200 Euro for 2020 for the palliative care advisory team. Costs per patient per day decreased significantly for 2020 compared to 2019 for the palliative care unit (*p*-value < 0.001) and increased significantly for the palliative care advisory team (*p*-value < 0.001). The share of overhead costs is accounting for more than two thirds of total costs. Whereas the overhead costs were significantly different (*p*-value = 0.000) for both settings (see Table 4, for detailed analyses of each professional group see supplementary Table 1, Appendix 2). Sensitivity analyses were conducted with increased nursing minutes, resulting in slightly significant direct unit-costs for the palliative care unit (*p*-value = 0.003), but not for the palliative care advisory team (*p*-value < 0.001, see supplementary Table 2, Appendix 3).

**Table 4:** Summary results costs, per patient, per day

	**Palliative care unit**			
	**2019**	**2020**	**growth 2019—2020 in %**	***p*-value**
Care				
Cases	55	76	38.2%	
Days ^a^	569	686	20.6%	
Length of stay	10.3	9.0	-12.8%	.536
Costs in €				
Total unit costs	1075.8	944.1	-12.2%	**.000**
Direct costs ^b^	134.7	131.3	-2.5%	.376
Overhead costs ^c^	941.1	812.8	-13.6%	**.000**
Share overhead ^d^	87.5%	86.1%	-1.6%	
	**Palliative care advisory team**			
	**2019**	**2020**	**growth 2019—2020 in %**	***p*-value**
Care				
Cases	135	120	-11.1%	
Days ^a^	851	645	-24.2%	
Length of stay	6.3	5.4	-14.7%	.422
Costs in €				
Total unit costs	161.4	200.8	24.4%	**.000**
Direct costs ^b^	54.4	57.3	5.3%	.540
Overhead costs ^c^	107.0	143.5	34.1%	**.000**
Share overhead ^d^	66.3%	71.5%	7.8%	

^a^Sum of nursing days (case multiplied by lenght of stay)\

^b^costs of resources in Euros per day

^c^costs of residual labor time, supplies and general hospital costs, per day

^d^Overhead costs/Total unit costs, *p*-values calculated with Mann–Whitney-U-tests

## Discussion

This is to our knowledge the first study quantitively describing the impact of the COVID-19 pandemic on work processes, time resources and costs in palliative care. The presented data indicate some minor differences before and during the pandemic and suggest changes in a) patient and care characteristics, b) work processes on team level, and c) costs per patient and day.

### Impact on patient and care characteristics

The data indicate some similarities and differences suggesting changes in care processes caused by the pandemic and the accompanying regulations. The proportion of people who died on the palliative care unit was almost identical in both years. The length of stay during the pandemic was shorter, both in the advisory team and the PCU. In contrast to the palliative care unit, less patients died when cared for by the palliative care advisory team during the pandemic time frame. This can be explained by the higher inhouse referral rate to the palliative care unit as the unit did not take any referrals from other hospitals in that time. With more available bed capacities patients could be transferred quicker from the advisory team to the palliative care unit.

The patients’ burden and palliative care needs as assessed by the IPOS indicate significant differences in some respects comparing the years in both settings. However, considering the rather high and differing numbers of patients with no completed IPOS assessment in both settings, IPOS scores are only comparable to a very limited extent. However, it is noticeable that the number of incomplete assessments decreased on the palliative care unit and increased in the palliative care advisory team. The increasing numbers of completed assessments on the palliative care unit can be explained by the ongoing implementation process of the use of outcome measures in the department and should be seen independently from the pandemic circumstances. However, for the palliative care advisory team a connection between the pandemic and the increase in incomplete assessments can be assumed with 7% more incomplete assessments in psychosocial items. This might be related to the significantly less documented staff minutes spent on contact with family and friends leading to the team’s inability to assess family burden and other psychosocial aspects of the patients.

### Impact on work processes on team level

Despite regulations to reduce infection risk, the number of professions involved in care and the cumulative time spent visiting patients did not decrease in 2020 compared with the previous year. However, data indicate that the visiting restrictions impacted on daily work routines. While the documented labour minutes per day and patient did not vary, a shift in activities could be observed. In both settings, less time was spent on contacts with families. In the palliative care advisory team, the decrease was in favour of contacts with other professionals, on the palliative care unit in favour of the minutes documented for direct patient contact. This is most probably related to the visiting restrictions, as families and friends were not allowed to come to the hospital and thus, direct meetings of professionals with relatives were not possible. On the palliative care unit, where limited visits were allowed, relatives wanted to use this time primarily with the patients and did not meet health care professionals. The impact of visiting restrictions becomes especially evident when looking at the shift in documented nursing times per field of activity. Family members are an important resource in palliative care since they provide emotional support and take on care activities themselves, such as helping the patient with body hygiene, use of the toilet, and helping with food preparation and intake. The lack of emotional and hands-on support caused by the contact restrictions needed to be compensated by nursing staff. Time spent on general nursing activities was not documented and accordingly not subject of this analysis. Accordingly, the calculated times and costs can only be interpreted in terms of a tendency, and it cannot be ruled out that there is a difference in nursing minutes per day and patient between the years and that the time spent in direct patient contact is actually much higher during the pandemic. This assumption is supported by the palliative care staffs’ perception of changes in their responsibilities and increased workload as reported in other studies [4, 6].

### Impact on cost per patient and day

Even though only slight differences in the overall documented staff time and length of care episodes can be observed, the cost calculation indicates that the pandemic also had an impact on the cost per day and patient. Concerning the unit-costs, total unit-costs differed significantly both for the palliative care unit and the palliative care advisory team, but in different directions. For the palliative care unit, the cost decreased while the cost increased in the palliative care advisory team. Personnel costs were the major cost component. This is in line with the findings of Gardiner et al. and Becker et al. [11, 12]. The differences were mainly due to the differing length of stay. Vogl et al. also identified a high correlation between unit-costs and length of stay, as well as personnel costs as main cost component [13]. The overhead costs were significantly different for both settings because these costs depend on the nursing days and hence on the length of stay, due to different capacity utilizations. The rising number of patients resulted in decreasing overhead costs and subsequently reduced total costs for the palliative care unit. In contrast, the nursing days decreased for the palliative care advisory team with rising overhead costs and subsequently higher total costs. Length of stay was chosen for allocation of overhead costs as it reflects the every-day care needs linked to them, and as they are often used in hospital cost allocation [13, 14]. A disadvantage is that allocating overheads by length of stay induces a high correlation with the unit-costs. Due to these overhead costs, the influence of COVID-19 on the costs of the palliative care unit and the palliative care advisory team depended strongly on the length of stay. The direct unit-costs per patient per day were not affected by the COVID-19 pandemic because the minutes per patient and day were equal every day. Also, the sensitivity analysis with increased nursing minutes showed only little impact on the direct unit-costs per patient per day. Hence, the direct costs per patient per day were comparable, regardless of the COVID-19 pandemic.

#### Clinical, political, and research implications

The shift in nursing time spent on contact to family on the palliative care unit during the pandemic underlines the important multifaceted role of nurses in care situations in general and during a crisis such as the pandemic specifically since they are the team members most likely compensating for the restricted access of visitors. According to a recent review by Hugelius, visitor restrictions have mainly negative effects in several medical fields, especially for patients and their families and increase therefore the burden for health care workers [15]. Health care workers are affected by the pandemic on two levels – on a personal level, as individuals meeting the same challenges any person meets during the time of uncertainty of this crisis, and on a professional level, since the pandemic affects their everyday work. High emotional distress and anxiety among health care workers in palliative care settings worldwide is the consequence [4, 5].

Contact restrictions also have an impact on the everyday life of patients and their families and affect the experience of illness and possible care decisions. This can result in palliative care patients being isolated or alone at the end of their lives or avoiding admission to the inpatient area for fear of infection and isolation. In addition, patients and their relatives can no longer decide freely about the palliative medical approach at the end of life [16, 17]. These circumstances often are accompanied by psychological and social problems [18]. Contact restrictions and the accompanying lack of personal contact with the patient and information on the situation are most likely also of consequence for the family and their ability to process the progress of an incurable disease. Families’ increased burden and emotional distress were reported because of visiting restrictions and the lack of information [19]. This may result in more complicated grief and feelings of guilt about not being able to care for their loved one.

Since contact to family members and friends was strictly limited, different aspects of the patients’ burden, such as unsolved problems with a family member, could easily be missed. In a regular palliative care setting, the staff would also talk to the family members and probably find other aspects restraining the patient. When this important part is missing, the care for the patient and support for the family fall short which might have consequences for the quality of care being provided during the time of the pandemic.

Previous literature investigates mainly qualitatively, with semi-structured questionnaires and interviews, the situation of palliative care in general. Hence, this analysis applies, in contrast to the existing literature, several standardized assessments for the comparison of patient characteristics. Data is collected in routine care to mirror the real world at a university hospital during the COVID-19 pandemic. Additionally, resources in minutes are collected at patient-level for diverse occupational groups, to allow a detailed analysis of differences between patients treated during and after the COVID-19 pandemic. Literature about the financial impact is very scarce. Changes in the daily routines, the higher workload and the visitor restrictions may subsequently increase the use of personnel resources and hence the costs for each patient. These assumptions about increasing costs should be investigated with empirical data to strengthen the mainly qualitative literature about increasing use of resources and costs.

Researchers performing health service research during a pandemic need to bear in mind the many effects the pandemic (or any other crisis) will have on the object of research, the field and accordingly the collected data. Care processes and quality of care might vary and have different outcomes during a crisis, and results need to be interpreted accordingly. Also, from a research ethics point of view researchers need to consider the increased burden of staff, families and patients and weigh up whether it is appropriate to add to this burden by conducting a research project in these exceptional circumstances.

## Strengths and limitations

This study has both strengths and limitations. One strength is the analyses of staff time and costs and not only patient characteristics. This was possible, as the staff of the Department of Palliative Medicine routinely documents patient related time. The main limitation is the use of routine data rather than data specifically collected for a research project. As described above, not all staff times were documented on the palliative care unit. This was especially the case for nurses, but it cannot be excluded that this also happened with other professional groups. Hence, the used data is incomplete and cannot be understood as the actual times spent for patient care. Differences between the observation periods might be more pronounced, if we would have been able to include all times spent on patient care on the palliative care unit.

Further, we conducted an exploratory analysis only based on routine data of one university hospital in Germany. Accordingly, the reported results mirror the work processes of this specific hospital against the background of the German health care system. Results might have been different if we could have included other institutional and country data. However, it can be expected that outcomes would be similar in other teams and countries since hygiene and contact regulations were not German specific as are the palliative care specific work processes such as the involvement of family members in care.

The cost calculation was a first attempt to capture the impact of COVID-19 on the costs of palliative care units and palliative care advisory teams. Due to the applied cost calculation method, it was possible to consider the resources in minutes for each patient. Additionally, the indirect costs were included in the analyses. Nevertheless, costs were collected from the accounting department with data from 2019. Hence, supplies and general hospital costs did not cover additional costs for example for COVID-19 tests and protection equipment. Whether there may be a bias, the costs would be underestimated for both settings. Furthermore, it was assumed that the number of employees and the payroll costs for the staff were equal for 2019 and 2020. If this was not the case, the overhead costs may be biased, resulting in either over- or underestimated unit-costs. The results of total unit-costs depend highly on the length of stay because the share of overhead costs is very high. Hence, the results are biased towards the degree of capacity utilization and the length of stay. Hence, this analysis is not appropriate for budget negotiations or for the development of a reimbursement system.

While a similar costing scheme has been used previously to calculate palliative care costs in the same university hospital [12], it is not possible to use these results as a pre-COVID-19 reference. Especially, this is due to substantial changes involving incomparable differences both in organizational units, and in personnel intensity, both key in the calculations.

## Conclusions

The COVID-19 pandemic and its accompanying regulations affected palliative care, especially in terms of work processes and total unit costs. While total unit-costs per patient per day differed significantly between the years, this difference was due to the cost dependency on length of stay associated overhead costs. Direct costs per patient per day, however, were comparable, regardless of the COVID-19 pandemic. Care processes and quality of care might, however, vary and have different outcomes during a crisis such as the COVID-19 pandemic.

Visiting restrictions impacted on daily work routines. While the documented labour minutes per day and patient did not vary, a shift in activities could be observed. Due to contact restrictions, time spent on contacts with families decreased. Since contact to family members and friends was strictly limited, different aspects of the patients’ burden, such as unsolved problems with a family member, could easily be missed which might have consequences for the quality of care being provided during the time of the pandemic.

## Supplementary Information

**Additional file 1: Appendix 1.** Boxplots on documented mean minutes spent on patientcare in 2019 and 2020.

**Additional file 2:**  **Appendix 2-3**.

## Data Availability

The datasets generated and/or analysed during the current study are not publicly available due the study site’s restrictions on data sharing but are available from the corresponding author on reasonable request.

## References

[1] Nouvet E, Sivaram M, Bezanson K, Krishnaraj G, Hunt M, de Laat S (2018). Palliative care in humanitarian crises: a review of the literature. J Int Humanit Act.

[2] Dunleavy L, Preston N, Bajwah S, Bradshaw A, Cripps R, Fraser LK, et al. “Necessity is the mother of invention”: Specialist palliative care service innovation and practice change in response to COVID-19. Results from a multinational survey (CovPall). Palliat Med. 2021;35(5):814–29. 10.1177/02692163211000660 PMC8114457 33754892

[3] Mitchell S, Oliver P, Gardiner C, Chapman H, Khan D, Boyd K (2021). Community end-of-life care during the COVID-19 pandemic: findings of a UK primary care survey. BJGP Open..

[4] Nestor S, O’Tuathaigh C, O’Brien  T (2021). Assessing the impact of COVID-19 on healthcare staff at a combined elderly care and specialist palliative care facility: a cross-sectional study. Palliat Med..

[5] Pastrana T, De Lima L, Pettus K, Ramsey A, Napier G, Wenk R (2021). The impact of COVID-19 on palliative care workers across the world: A qualitative analysis of responses to open-ended questions. Palliat Supp Care.

[6] Sleeman KE, Cripps RL, Murtagh FE, Oluyase AO, Hocaoglu MB, Maddocks M (2022). Change in Activity of Palliative Care Services during the Covid-19 Pandemic. J Palliat Med..

[7] von Elm E, Altman DG, Egger M, Pocock SJ, Gøtzsche PC, Vandenbroucke JP (2014). The Strengthening the Reporting of Observational Studies in Epidemiology (STROBE) Statement: guidelines for reporting observational studies. Int J Surg (London, England).

[8] Eagar K, Green J, Gordon R (2004). An Australian casemix classification for palliative care: technical development and results. Palliat Med.

[9] Murtagh FE, Ramsenthaler C, Firth A, Groeneveld EI, Lovell N, Simon ST, et al. A brief, patient- and proxy-reported outcome measure in advanced illness: Validity, reliability and responsiveness of the Integrated Palliative care Outcome Scale (IPOS). Palliat Med.0(0):0269216319854264. 10.1177/0269216319854264 PMC6691591 31185804

[10] Mosoiu D, Dumitrescu M, Connor SR (2014). Developing a costing framework for palliative care services. J Pain Symptom Manage.

[11] Gardiner C, Ingleton C, Ryan T, Ward S, Gott M (2017). What cost components are relevant for economic evaluations of palliative care, and what approaches are used to measure these costs? A systematic review. Palliat Med.

[12] Becker C, Leidl R, Schildmann E, Hodiamont F, Bausewein C (2018). A pilot study on patient-related costs and factors associated with the cost of specialist palliative care in the hospital: first steps towards a patient classification system in Germany. Cost Eff Resour Alloc.

[13] Vogl M, Schildmann E, Leidl R, Hodiamont F, Kalies H, Maier BO (2018). Redefining diagnosis-related groups (DRGs) for palliative care - a cross-sectional study in two German centres. BMC Palliat Care.

[14] (InEK). IfdEiK. Kalkulation von Behandlungskosten Version 4.0 Düsseldorf2016 Available from: https://www.g-drg.de/Kalkulation2/DRG-Fallpauschalen_17b_KHG/Kalkulationshandbuch.

[15] Hugelius K, Harada N, Marutani M (2021). Consequences of visiting restrictions during the COVID-19 pandemic: an integrative review. Int J Nurs Stud..

[16] Arya A, Buchman S, Gagnon B, Downar J (2020). Pandemic palliative care: beyond ventilators and saving lives. CMAJ..

[17] Organization WH (2018). Integrating palliative care and symptom relief into responses to humanitarian emergencies and crises: a WHO guide.

[18] Krakauer E, Daubman B, Aloudat T, Bhadelia N, Black L, Janjanin S (2020). Palliative care needs of people affected by natural hazards, political or ethnic conflict, epidemics of life-threatening infections, and other humanitarian crises.

[19] Pauli B, Strupp J, Schloesser K, Voltz R, Jung N, Leisse C, et al. It’s like standing in front of a prison fence - Dying during the SARS-CoV2 pandemic: A qualitative study of bereaved relatives’ experiences. Palliat Med. 2022;36(4):708–16. 10.1177/02692163221076355 35350933

