# Correction to: The impact of the COVID-19 pandemic on processes, resource use and cost in palliative care

**DOI:** 10.1186/s12904-023-01178-5

**Published:** 2023-05-10

**Authors:** Farina Hodiamont, Caroline Schatz, Eva Schildmann, Zulfiya Syunyaeva, Katerina Hriskova, Constanze Rémi, Reiner Leidl, Susanne Tänzler, Claudia Bausewein

**Affiliations:** 1 Department of Palliative Medicine, University Hospital, LMU Munich, Munich, Germany; 2 Helmholtz Zentrum München, Institute of Health Economics and Health Care Management, Munich, Germany; 3 Ludwig-Maximilians-Universität München, LMU Munich School of Management, Institute of Health Economics and Health Care Management, Munich, Germany; 4 Charité ? Universitätsmedizin Berlin, corporate member of Freie Universität Berlin and Humboldt-Universität Zu Berlin, Department of Hematology, Oncology and Cancer Immunology, Oncological Palliative Care & Charité Comprehensive Cancer Center, Berlin, Germany; 5 Charité Universitätsmedizin Berlin, Department of Pediatric Respiratory Medicine, Immunology and Critical Care Medicine and Cystic Fibrosis Center, Berlin, Germany

**Correction: BMC Palliat Care 22, 36 (2023).**

10.1186/s12904-023-01151-2

Following publication of the original article [1], the authors reported that there was an added section in the article. The following section should not be part of the article.

Authors’ information

All authors belong to the EAST team in the Institute of Plasma Physics, Chinese Academy of Sciences.

The original article has been corrected.
