# Supplementary material for: The impact of the COVID-19 pandemic on processes, resource use and cost in palliative care
Source: BMC Palliat Care. 2023 Apr 6;22:36. doi: 10.1186/s12904-023-01151-2 (PMC10077306; doi:10.1186/s12904-023-01151-2)

**Appendix 1: Boxplots on documented mean minutes spent on patient care in 2019 and 2020**

**Palliative care unit**


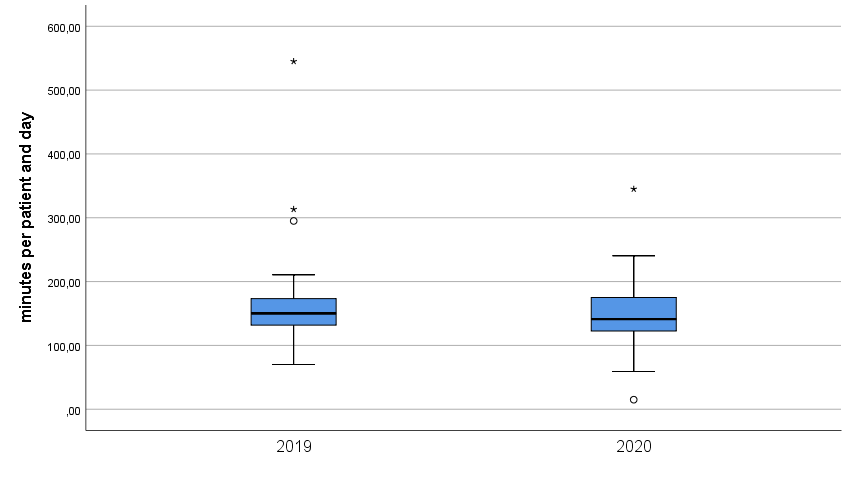


**Palliative care advisory team**


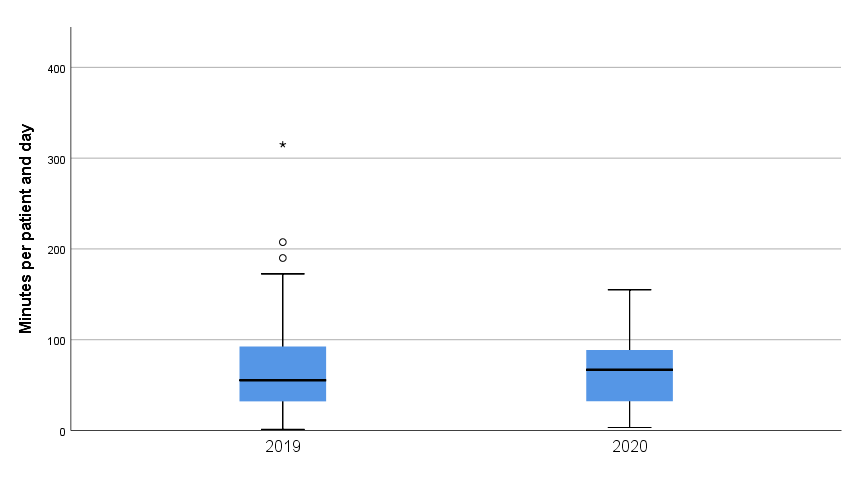

Supplement: Supplementary file 1 — Additional file 1: Appendix 1. Boxplots on documented mean minutes spent on patientcare in 2019 and 2020. [file 12904_2023_1151_MOESM1_ESM.docx]
