# Supplementary material for: The impact of the COVID-19 pandemic on processes, resource use and cost in palliative care
Source: BMC Palliat Care. 2023 Apr 6;22:36. doi: 10.1186/s12904-023-01151-2 (PMC10077306; doi:10.1186/s12904-023-01151-2)
Supplement: Supplementary file 2 — Additional file 2: Appendix 2-3. [file 12904_2023_1151_MOESM2_ESM.docx]

**Supplementary material**

**1. Detailed analyses of unit-costs per patient per day for each professional group**

Supplementary Table 1 shows the total unit-costs and the direct- and indirect costs for each professional group per patient, per day. Since nursing minutes were the highest for the palliative care unit, direct nursing costs were also the most among the direct costs. Although, physician minutes were approximately one third of the nursing minutes, the direct costs for physicians were more than a half of the direct nursing costs. This was the result of the cost rates because physicians had higher salaries than nurses. The main proportion of the total costs were the overhead costs for personnel (physicians, nurses and other), followed by general hospital costs.

In contrast, for the palliative care advisory team the minutes for the physicians were higher than the nursing time. To mention, that only the palliative care nursing minutes were documented, no general care (for example no basic care at the oncological ward). Hence, the direct costs for physicians were the highest per patient, per day. The indirect costs consisted mainly of personnel overhead costs, followed by the general hospital costs, similar to the palliative care unit.

**2. Sensitivity analysis**

Due to the high importance of nursing, but the relatively less documented minutes, a sensitivity analysis, with increasing the direct nursing minutes by one standard deviation, was calculated. Supplementary Table 2 indicates that the higher the direct documentation the more likely the significance of direct costs. The overhead costs could be shifted to direct costs and then the results would change. Hence, whether the documentation could be improved, the difference for patients treated during the COVID-19 pandemic compared to patients treated before could be significant. Although, these univariate tests did not account for covariates.
